# Retraction Note: Novel green synthesis and antioxidant, cytotoxicity, antimicrobial, antidiabetic, anticholinergics, and wound healing properties of cobalt nanoparticles containing *Ziziphora clinopodioides* Lam leaves extract

**DOI:** 10.1038/s41598-020-71843-9

**Published:** 2020-09-07

**Authors:** Huifang Hou, Behnam Mahdavi, Sogand Paydarfard, Mohammad Mahdi Zangeneh, Akram Zangeneh, Nastaran Sadeghian, Parham Taslimi, Vildan Erduran, Fatih Sen

**Affiliations:** 1 School of Basic Medicine, Xinxiang Medical University, Xinxiang, 453003 China; 2 Department of Chemistry, Faculty of Science, Hakim Sabzevari University, Sabzevar, Iran; 3 Biotechnology and Medicinal Plants Research Center, Ilam University of Medical Sciences, Ilam, Iran; 4 Department of Clinical Sciences, Faculty of Veterinary Medicine, Razi University, Kermanshah, Iran; 5 Department of Chemistry, Faculty of Sciences, Ataturk University, 25240 Erzurum, Turkey; 6 Department of Biotechnology, Faculty of Science, Bartin University, 74100 Bartin, Turkey; 7 Sen Research Group, Biochemistry Department, Faculty of Arts and Science, Dumlupinar University, Evliya Celebi Campus, 43100 Kutahya, Turkey

Retraction of: *Scientific Reports* 10.1038/s41598-020-68951-x, published online 22 July 2020

The Editors have retracted this article.

After publication it was brought to the Editors' attention that some of the figures in this article contain images duplicated from other sources. Specifically:

- Figure 2 appears to be duplicated (with modification to contrast and 90 degrees rotation) from Figure 1 in Rouhollahi et al.^1^, which reports a different set of experiments.
- Panels in Figure 9 appear to be duplicated from panels in Figure 1 in Ghashghaii et al.^2^, which reports a different set of experiments.
- Figure 10 contains internal duplications between panels A and B, and between panels E and F, which all represent different experimental conditions.

In addition, Figure 1 appears to have been reused (with alterations to the aspect ratio) from an online source^3^.

The Editors therefore no longer have confidence in the integrity of the data presented.

Behnam Mahdavi, Sogand Paydarfard, Mohammad Mahdi Zangeneh, Akram Zangeneh, Nastaran Sadeghian, Parham Taslimi and Fatih Sen do not agree with this retraction. Huifang Hou and Vildan Erduran have not responded to any correspondence from the Editors about this retraction.
